# Life history characteristics of birds influence patterns of tick parasitism

**DOI:** 10.1080/20008686.2018.1547096

**Published:** 2018-11-27

**Authors:** R. Jory Brinkerhoff, Lena Dang, Henry M. Streby, Maren Gimpel

**Affiliations:** a Department of Biology, University of Richmond, Richmond, VA, USA; b School of Life Sciences, University of KwaZulu-Natal, Pietermaritzburg, South Africa; c Department of Fisheries, Wildlife, and Conservation Biology, University of Minnesota, St. Paul, MN, USA; d Department of Environmental Sciences, University of Toledo, Toledo, OH, USA; e Forman’s Branch Bird Observatory, Washington College, Chestertown, MD, USA

**Keywords:** *Ixodes scapularis*, avian ecology and life history, parasitism, zoonotic disease

## Abstract

**Introduction:** Birds serve as reservoirs for tick-borne pathogens as well as hosts for multiple tick species of public health relevance.  Birds may perpetuate life cycles of vectors and vector-borne pathogens and disperse disease vectors over long distances, supplementing populations at range margins or seeding invading populations beyond the edges of current tick distributions.  Our goal for this study was to identify life history characteristics of birds that most strongly affect tick parasitism.

**Materials and Methods:** We collected 6203 ticks from 5426 birds from two sites in eastern North America and used field-derived parasitism data and published literature to analyze impacts of life history factors on tick parasitism in birds.

**Results and Discussion:** We identified body size and nest site to have the strongest impact on tick prevalence and abundance in the songbird species included in this study.  Our findings reveal site-independent patterns in tick parasitism on birds and suggest that physical more than behavioral characteristics may influence a bird species’ suitability as a host for ticks.

**Conclusions:** The data and results published here will contribute to a growing body of literature and information on bird-tick interactions and will help elucidate patterns of tick and tick-borne pathogen geographic expansion.

## Introduction

Ticks serve as hosts for a large number of zoonotic pathogens and the relative importance of birds in supporting tick life cycles and as reservoirs for vector-borne pathogens has gained attention of recent years [1–3]. Bird migration and dispersal have been implicated in the spread of tick-transmitted pathogens in Europe and North America [4–8] and potentially serve as a common mechanism for range expansion of vector-borne pathogens [2]. Migratory movement of bird-associated *Ixodes scapularis* ticks infected with the Lyme disease agent, *Borrelia burgdorferi*, may explain recently observed northward and southward geographic expansion of human Lyme disease cases [1,3,5,8–12, 13] and a theoretical mathematical analysis demonstrated that deposition of ticks by migrating birds can enhance tick population growth and thus increase human risk of tick-borne disease [14]. Moreover, the recent establishment of the disease vector long-horned tick, *Haemaphysalis longicornis*, in New Jersey, USA, and its subsequent detection in a number of other states underscores the rapidity with which our understanding of geographic distributions of ticks can change [15]. *H. longicornis* is known to parasitize birds [e.g. 16], and its congener *H. leporispalustris* is a common bird parasite [e.g. 17], suggesting bird mediated range expansion of this species in North America is possible.

Notwithstanding the potentially important role of birds in driving range expansion of tick-transmitted disease, birds may also be involved in enzootic maintenance of tick-borne pathogens. For example, the detection of infected larval ticks with *B. burgdorferi* from avian hosts strongly suggests that birds are capable of, and potentially important to, the transmission of this pathogen in natural systems [18–20]. Moreover, many of the *B. burgdorferi* genotypic variants detected in birds have been previously unreported, suggesting that bird-tick transmission cycles may contribute to the maintenance of genetic variation in this pathogenic bacterium [9,19,21]. Although certain small mammal species are likely to be most important in terms of enzootic transmission and maintenance of this pathogen [22], birds may play an important role in the evolutionary ecology and long-distance movement of B. burgdorferi [3].

Laboratory transmission experiments indicate that certain North American bird species are highly competent reservoirs for *B. burgdorferi* infection [eg American Robin, 23] whereas others may be substantially less capable of becoming infected with this agent [eg Gray Catbird, 24]. Reservoir competence for *B. burgdorferi* may be strain/genotype dependent [25], but field-derived estimates of competence are generally consistent with laboratory quantification of reservoir competence. For example, Brinkerhoff et al. [1],reviewed a large number of papers reporting *B. burgdorferi* infection in bird-derived *I. scapularis* larvae and found substantial heterogeneity in rates of tick parasitism as well as infection prevalence in ticks. In that meta-analysis, very few infected ticks were recovered from Gray Catbirds whereas American Robins had one of the highest rates of producing B. burgdorferi-infected *I. scapularis* larvae. More recently, Loss et al. [3] quantified the contributions of individual tick species to tick- and pathogen maintenance cycles and concluded that migration and foraging strategies may interact to contribute to high tick infestation on particular bird species. One limitation of pooled or meta-analyses is that the consolidation of data from multiple studies represents different sampling sites and conditions, inconsistent methodologies and sampling seasonality, and obscures effects of time when data are pooled among years or even decades. Moreover, ecological processes are variable in space; for example, the extent to which similar factors drive Lyme disease risk and population expansion of blacklegged ticks between the northeastern and Midwestern Lyme disease foci is not clear [26].

Although the recovery of *B. burgdorferi*-infected larval ticks from a multitude of North American songbird species has been previously reported [e.g. 27, 19], the drivers of heterogeneity among bird species in tick parasitism are less well understood. Just as all individuals of a given species, and all species in an assemblage of hosts have unequal parasite or pathogen burdens [28], all bird species are not equally likely to acquire ticks or become infected with zoonotic agents. Heterogeneity within and among species in patterns of parasitism is a common feature of most host-parasite systems where a small proportion of individuals within populations, or a small number of species within a community, supports a disproportionately large number of parasites; in fact, this overdispersion may facilitate the persistence of both parasite and host [29]. Heavily parasitized entities may serve as superspreaders and be responsible for the perpetuation of parasite populations and play a large role in disease transmission [30]. The importance of susperspreaders in disease transmission dynamics has been the target of recent research, but mechanisms to account for differential parasite/pathogen burden are complex and often poorly understood (Paull et al. 2012). Life history characteristics of birds are known to affect the occurrence of internal and external parasites of mammals [31] and the occurrence of internal parasites in birds may be driven by life history characteristics. Recently, life history drivers of tick occurrence on birds were identified in European systems [27,32] and studies in North America have also begun to elucidate aspects of bird ecology and life history that may impact parasitism by ticks and bird-mediate tick range expansion [3,8]. Our goal was to determine if there is consistency among geographically distant Lyme disease foci in patterns of tick parasitism on birds and if certain host characteristics are associated with high tick parasitism. We were specifically interested in the effects of bird body mass and aspects of habitat use and behavior. We expected to find that species with larger average body mass would have a greater tick burden and also expected to find higher tick burdens on bird species with low-level nesting and/or foraging behavior since ixodid ticks do not typically quest higher than 1.5 m from the ground [e g. 33]

## Materials and methods

**Figure 1.**
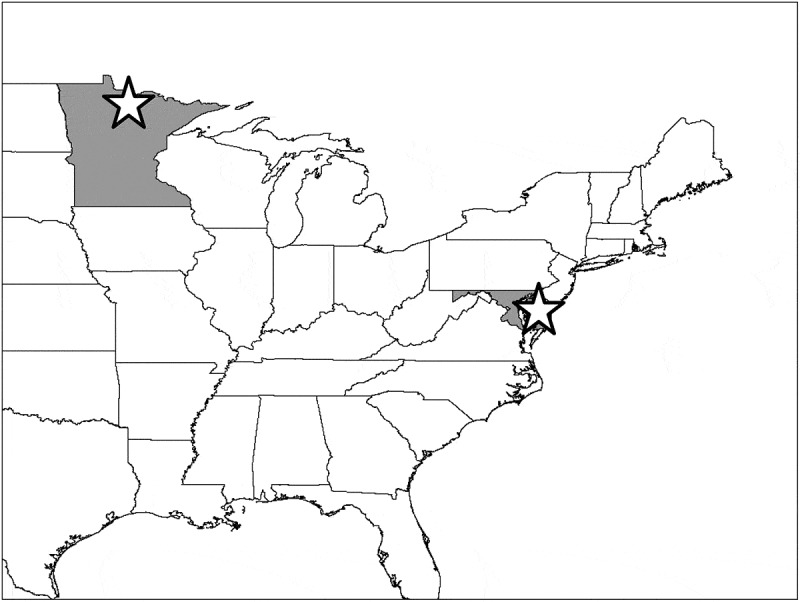
Locations of field sampling locations (open stars) in Minnesota and Maryland (shaded polygons).

We collected ticks from migratory and resident songbirds at two primary field sites (eastern Maryland and northern Minnesota, Figure 1) in 2008 and 2009. The Maryland field site, adjacent to the Chester River and consisting mostly of a rural mosaic of row-crop agricultural fields and both upland and wetland early successional woodlots, was sampled from March to May and August through November in each year of the study (USGS permit no. 21885, held by James Gruber). The Minnesota site, consisting primarily of early successional deciduous forest stands (ie regenerating clearcuts) near edges of mature deciduous and mixed deciduous-coniferous forest habitat, was sampled from May through September (USGS permit no. 21631, held by D.A. Andersen). All birds were sampled with Japanese mistnets and thoroughly inspected for ticks. Standard biometric data including sex, age, wing chord, body condition, and weight were collected before each bird was released. At the Minnesota site, up to 24 nets were run per day for approximately three hours after dawn for 6 days per week. Nets in Maryland (up to 100, weather and staff permitting) were run six days per week from late March through late early November with the greatest sampling intensity happening during migratory periods (ie 1 March – 31 May and 1 Aug – 30 November) and reduced effort in mid-summer months. As at the Minnesota field site, nets were open for roughly three hours each morning.

Ticks were collected in 2 ml screw-cap centrifuge tubes filled with 70% ethanol. All ticks were identified to life stage and species by light microscopy using dichotomous keys [34,35]. For each bird species at each site, we calculated prevalence (proportion of individuals parasitized by ticks) and abundance (total ticks collected divided by total number of individuals sampled) of all tick species combined. We used literature sources and databases [36,37] to assign average mass, nesting microhabitat, and feeding behavior for each species. We used a weighted regression model to determine if tick prevalence on a given bird species at the Maryland field site was predictive of prevalence at the Minnesota field site with weight values being proportional to the number of individuals for each species that was sampled and checked for presence of ticks. We ranked the relative importance of a given bird species as a tick host by summing all species-specific tick abundance values and then calculating the proportion of total tick abundance attributed to each species. Prevalence data were arcsine square-root transformed to improve residual diagnostics and analyzed with a linear model. Abundance data were analyzed with a general linear model using a quasi-Poisson error distribution and a log link. Because there were only three birds in the cavity nesting group, two of which had very high tick parasitism levels, we analyzed the dataset with and without these species. All analyses were conducted using R [38]

## Results

**Figure 2.**
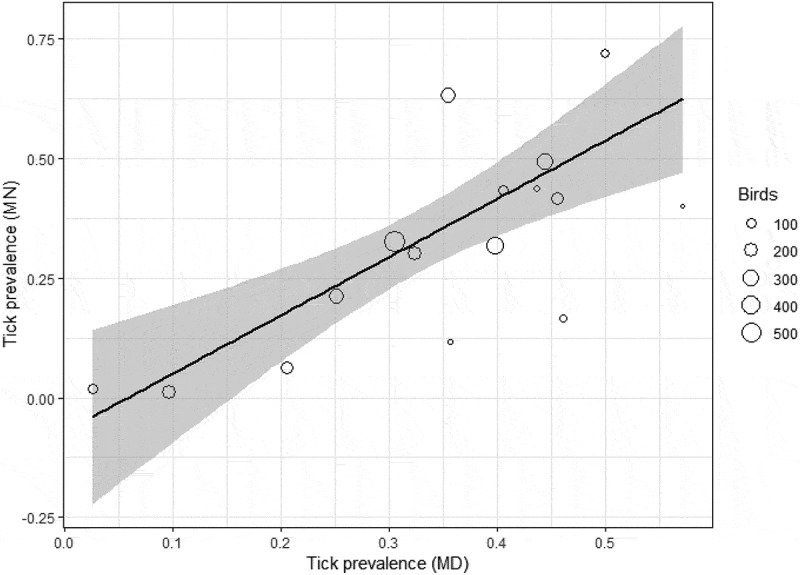
Tick prevalence in Minnesota on a subset of 16 bird species found at both sites as a function of tick prevalence on those same species in Maryland (F = 21.4, P = 0.0004, DF = 14, adjusted r^2^ = 0.58), weighted by sample size for each species (‘Birds’).

**Figure 3.**
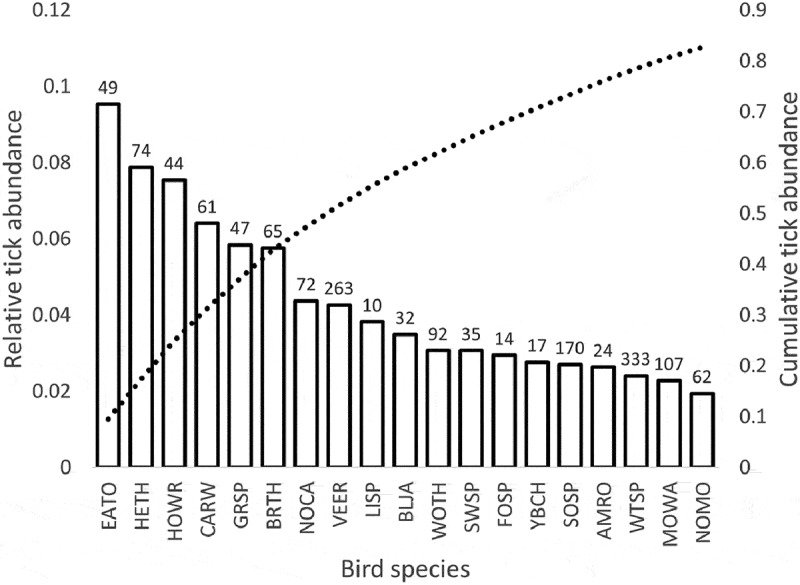
Ranked relative contribution of each bird species to the total number of ticks from all species (left vertical axis), as well as the cumulative abundance of ticks collected from all bird species, which crosses 50% with the eighth species (VEER; dashed line, right vertical axis). Tick abundance values for each species (calculated as the total number of ticks divided by the total number of individuals) were summed and the relative abundance was calculated for each species (tick abundance on species *i* divided by summed tick abundance). Numbers above bars represent total sample size for each species pooled across both sampling locations.

We collected a total of 6,203 ticks, representing six species, from 5,426 individual birds, representing 64 species Table 1; Table S1) with the most abundant ticks at both sites being *Ixodes scapularis* and *Haemaphysalis leporispalustris* (Table 2).  Table 1, Table S1). All bird species fell into one of two foraging classes (ground versus foliage/tree) and into one of four nesting microhabitats (ground, shrub, tree, and cavity). Bird species richness and diversity and tick species richness and diversity were higher at the Maryland site than at the Minnesota site (Table 1, Table 2). After removing bird species that were encountered fewer than 10 times, we were left with 50 bird species, 5370 individual birds, and 6091 ticks for analysis. For species that were encountered at least 10 times at each field site (n = 16), we found that tick prevalence at the Minnesota field site was significantly related to tick prevalence at the Maryland site for a given bird species (adjusted r^2^ = 0.58, F = 21.4, DF = 14, P = 0.0004) (Figure 2). The majority of all ticks collected in this study came from only eight bird species and the least parasitized 50% of bird species in this study accounted for only 10% of all ticks we collected. After adjusting for sample size, we found that the eight most heavily parasitized species, or those with the highest relative tick abundance, accounted for over half (51.6%) of all ticks we collected (Figure 3).

**Table 1.** Number and richness of bird and tick species at the Maryland and Minnesota study sites.

Field site	Total birds sampled	Bird species sampled for ticks	Total ticks collected	Tick species richness
Chestertown, MD	2608	53	3641	6
Cass and Itasca Counties, MN	2818	33	2562	2

**Table 2.** Numbers of immature *Ixodes scapularis* and *Haemaphysalis leporispalustris* collected from birds at each study site. Additional tick species recorded at the Maryland site included *Amblyomma americanum* (N=29), A. *maculatum* (N=129), *Ixodes brunneus* (N=45) and I. *dentatus* (N=178) with an additional six unidentified ticks.

Site	*I. scapularis* collected	*H. leporispaulstris* collected
Chestertown, MD	844	2412
Cass and Itasca Counties, MN	1307	1253

**Figure 4.**
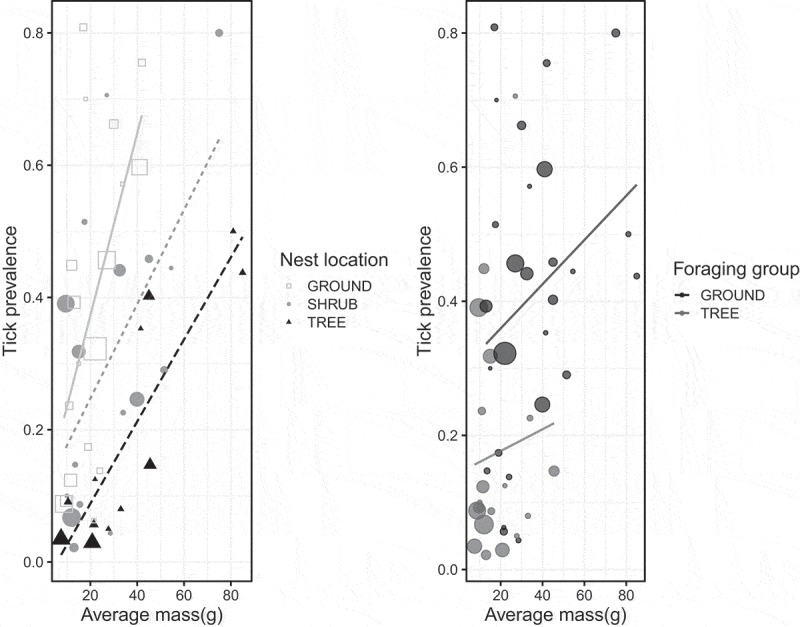
Tick prevalence as a function of average mass for a given bird species, coded by nesting location (ground versus shrub versus tree, left) and coded by foraging group (ground versus tree, right). Cavity nesting birds (N = 3) were removed from these plots but did not affect the results of regression analysis. Data points are scaled relative to the number of observations per species.

**Figure 5.**
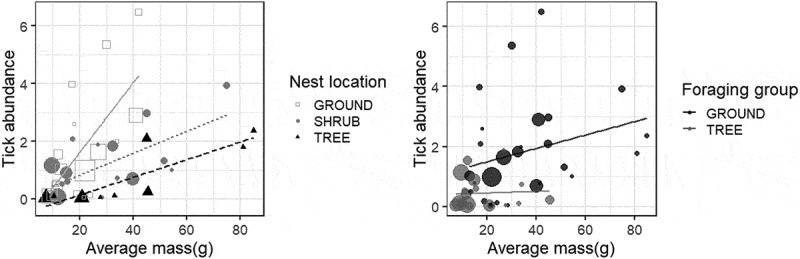
Tick abundance as a function of average mass for a given bird species, coded by nesting location (ground versus shrub versus tree, left) and coded by foraging group (ground versus tree, right). Cavity nesting birds (N = 3) were removed from these plots but did not affect the results of regression analysis. Data points are scaled relative to the number of observations per species.

Linear models revealed a significant effect of mass (F = 20.28, P < 0.0001, df = 1) and nesting site (F = 7.80, P = 0.0003, DF = 3) and a marginally significant effect of foraging category (ground versus tree, F = 3.61, P = 0.064, DF = 1) on tick prevalence (Figure 4). Because there were only three cavity-nesting species, two of which had high tick prevalence, we re-ran the analysis on a subset of the data with the cavity-nesting species excluded and found qualitatively similar results: mass and nest site were significantly predictive of tick prevalence (F = 24.24, P < 0.0001, DF = 1 and F = 11.88, P < 0.0001, DF = 2, respectively) while foraging location had a marginally significant effect on tick prevalence (F = 3.09, P = 0.086, DF = 1). Tick abundance was significantly positively affected by bird mass (F = 24.4, P < 0.0001, DF = 1) and nest site (F = 6.9, P = 0.0006, DF = 3), and marginally affected by foraging habitat (F = 3.23, P = 0.079, DF = 1) (Figure 5). These three variables accounted for 47% of the variation in tick abundance on birds. We note that the mass of ground-foraging birds was significantly higher (F = 19.19, p < 0.0001) than tree-foraging birds but that no such differences in mass were detected among the different nest site categories (F = 1.89, P = 0.14). However, ground-foraging birds were more likely than tree-foraging birds to be ground nesters as well.

## Discussion

Migratory hosts, such as many bird species, have the capacity to spread disease agents and vectors quickly across vast geographic areas [39]. Variation within and among species in their capacity to support parasites complicates our ability to predict patterns of parasite and pathogen range expansion via migratory hosts. The notion that a small proportion of superspreading individuals within a population is responsible for a disproportionate amount of pathogen transmission [29] has recently been expanded to address spatial variation in disease risk and heterogeneity among species within a community in pathogen and parasite prevalence and abundance [30]. Our goal was to identify characteristics of bird species that make them more likely to support, and potentially disperse, tick populations. Our results are consistent with the superspreader species hypothesis in that particular bird species are much more likely than others to encounter and/or become parasitized by ticks. Specifically, we demonstrated that 1) there is substantial heterogeneity among bird species in patterns of tick parasitism, 2) metrics of tick parasitism on a given bird species are largely consistent across space, and 3) specific bird life history characteristics are important predictors of tick parasitism on birds.

We found that there are apparently predictable patterns of tick prevalence on birds with the same species being relatively more or less heavily parasitized in both Maryland and Minnesota (Figure 2). Interestingly, these patterns did not seem to be driven by particular tick species; the number of *H. leporispaulstris* ticks was strongly correlated with the number of *I. scapularis* on a given bird species for the most commonly sampled birds among both study sites (P = 0.01, T = 2.96, DF = 14, r = 0.61). Taken together, these data support the hypothesis that there is heterogeneity among individuals within a species and among species within a community in parasite occurrence. These results also suggest that there are species-specific characteristics that are important drivers of tick parasitism on birds.

Bird foraging behavior, previously identified as a key driver of tick parasitism [reviewed in 2], was not a strongly significant predictor of tick prevalence or abundance after controlling for effects of body mass. In univariate analysis, ground foragers show significantly higher tick occurrence than do tree foragers (Figures 4 and 5) and the fact that the ground foraging birds in our study are significantly heavier than foliage-gleaners may be obscuring effects of behavior with effects of overall body size. As a result, we cannot conclude that foraging behavior, categorized as ground- versus tree-based foraging, is a significant predictor of tick parasitism or if simply being a larger-bodied bird, irrespective of amount of time spent near the ground, is the key determinant of tick acquisition. Newman et al. [40], suggested that larger birds have more surface area of exposed skin (ie around eyes and bills where many ticks on birds are found) and thus smaller body size may limit the number of ticks a bird can support. Interestingly, even after effects of body mass were accounted for, nesting microhabitat did emerge as a driver of tick prevalence and abundance. This result suggests, intuitively, that there are behaviors and/or patterns of habitat use that are important to tick parasitism. Given that ticks host-seek relatively close to the ground it is somewhat surprising that foraging behavior was not statistically associated with tick parasitism in this study. It is possible that nesting habitat is a better index of overall the time a bird spends near the ground than foraging behavior, at least for birds of similar body size. We also note that the life history traits investigated here are not independent of each other: larger birds tend to nest and forage on the ground and smaller birds are more likely to nest and forage in trees. Similarly, simple categorizations into nesting and foraging groups belie the complexity of bird behavior and variation within and among populations in habitat use and microhabitat selection for different behaviors. Certainly some of the observed variation in each response variable within a foraging or nesting group (Figures 4 and 5) results from imperfect classification of species into overly-simplified behavioral categories. However, caveats aside, our results are consistent with findings from European systems where bird body mass and foraging height in the canopy, a variable we did not measure, were among the strongest predictors of tick abundance on birds [32]. We also note that habitat use and associations for individual bird species, factors we did not measure or address, are important determinants of tick parasitism [41] with even urban species serving as potential dispersers of certain tick species [7].

Birds are commonly parasitized by ticks and have been implicated in the movement of tick-borne zoonotic pathogens, but so far, a relatively small (but growing) subset of studies studies have concentrated on the ecological and life history characteristics that affect patterns of tick parasitism [see and for recent reviews 2,3,]. Our goal was to determine if particular qualities of some bird species increase their likelihood of encountering and being parasitized by ticks and we found strong patterns within and among species in both tick prevalence and abundance. Specifically, we found that patterns of parasitism were largely consistent between the two sites we studied, suggesting that bird- rather than habitat-specific or regional patterns drive tick parasitism on birds. Moreover, we found strong dependence of both tick prevalence and abundance on bird body mass and nesting habitat with larger, ground-nesting birds most likely to have ticks and generally supporting larger numbers of ticks.
