# Supplementary material for: Life history characteristics of birds influence patterns of tick parasitism
Source: Infect Ecol Epidemiol. 2018 Nov 27;9(1):1547096. doi: 10.1080/20008686.2018.1547096 (PMC6263100; doi:10.1080/20008686.2018.1547096)
Supplement: Supplemental Material [file ZIEE_A_1547096_SM1731.docx]

| Field site | Total birds sampled | Bird species sampled for ticks | Total ticks collected | Tick species richness |
| --- | --- | --- | --- | --- |
| Chestertown, MD | 2608 | 53 | 3641 | 6 |
| Cass and Itasca Counties, MN | 2818 | 33 | 2562 | 2 |

Table 1. Number and diversity (calculated as Shannon’s index, H’) of bird and tick species at the Maryland and Minnesota study sites.

Table 2. Numbers of immature *Ixodes scapuarlis* and *Haemaphysalis leporispalustris* collected from birds at each study site.

| Site | *I. scapularis* collected | *H. leporispaulstris* collected |
| --- | --- | --- |
| Chestertown, MD | 844 | 2412 |
| Cass and Itasca Counties, MN | 1307 | 1253 |

Table S1. Numbers of birds sampled and ticks collected, as well as tick prevalence and average tick abundance for each species. Foraging behavior and nesting habitat data were taken from published resources (Ehrlich et al. 1992, Poole et al. 2005).

| Bird Species | Species Abbreviation | Individuals Sampled | Individuals with Ticks | Total Ticks Collected | Nest Habitat | Foraging Behavior | Tick Abundance | Tick Prevalence |
| --- | --- | --- | --- | --- | --- | --- | --- | --- |
| American Goldfinch | AMGO | 46 | 4 | 28 | SHRUB | FOL GLEAN | 0.61 | 0.09 |
| American Redstart | AMRE | 230 | 8 | 12 | TREE | FOL GLEAN | 0.05 | 0.03 |
| American Robin | AMRO | 24 | 12 | 43 | TREE | GROUND | 1.79 | 0.50 |
| Black-capped Chickadee | BCCH | 68 | 1 | 1 | CAVITY | FOL GLEAN | 0.01 | 0.01 |
| Black-throated Blue Warbler | BTBW | 20 | 2 | 2 | SHRUB | FOL GLEAN | 0.10 | 0.10 |
| Blue Grosbeak | BLGR | 23 | 1 | 1 | SHRUB | GROUND | 0.04 | 0.04 |
| Blue Jay | BLJA | 32 | 14 | 76 | TREE | GROUND | 2.38 | 0.44 |
| Brown-headed Cowbird | BHCO | 17 | 6 | 13 | TREE | GROUND | 0.76 | 0.35 |
| Brown Thrasher | BRTH | 65 | 52 | 255 | SHRUB | GROUND | 3.92 | 0.80 |
| Canada Warbler | CAWA | 55 | 13 | 30 | GROUND | FOL GLEAN | 0.55 | 0.24 |
| Carolina Wren | CARW | 61 | 49 | 266 | CAVITY | GROUND | 4.36 | 0.80 |
| Cedar Waxwing | CEDW | 25 | 2 | 3 | TREE | FOL GLEAN | 0.12 | 0.08 |
| Chestnut-sided Warbler | CSWA | 402 | 27 | 28 | SHRUB | FOL GLEAN | 0.07 | 0.07 |
| Chipping Sparrow | CHSP | 34 | 5 | 17 | SHRUB | GROUND | 0.50 | 0.15 |
| Common Yellowthroat | COYE | 361 | 141 | 418 | SHRUB | FOL GLEAN | 1.16 | 0.39 |
| Connecticut Warbler | CONW | 10 | 3 | 8 | GROUND | GROUND | 0.80 | 0.30 |
| Eastern Towhee | EATO | 49 | 37 | 318 | GROUND | GROUND | 6.49 | 0.76 |
| Field Sparrow | FISP | 163 | 64 | 169 | GROUND | GROUND | 1.04 | 0.39 |
| Fox Sparrow | FOSP | 14 | 8 | 28 | GROUND | GROUND | 2.00 | 0.57 |
| Golden-winged Warbler | GWWA | 130 | 12 | 15 | GROUND | FOL GLEAN | 0.12 | 0.09 |
| Grasshopper Sparrow | GRSP | 47 | 38 | 187 | GROUND | GROUND | 3.98 | 0.81 |
| Gray Catbird | GRCA | 252 | 62 | 178 | SHRUB | GROUND | 0.71 | 0.25 |
| Hermit Thrush | HETH | 74 | 49 | 397 | GROUND | GROUND | 5.36 | 0.66 |
| House Finch | HOFI | 53 | 3 | 3 | TREE | GROUND | 0.06 | 0.06 |
| House Wren | HOWR | 44 | 35 | 226 | CAVITY | FOL GLEAN | 5.14 | 0.80 |
| Indigo Bunting | INBU | 217 | 69 | 194 | SHRUB | FOL GLEAN | 0.89 | 0.32 |
| Lincoln's Sparrow | LISP | 10 | 7 | 26 | GROUND | GROUND | 2.60 | 0.70 |
| Magnolia Warbler | MAWA | 44 | 4 | 4 | TREE | FOL GLEAN | 0.09 | 0.09 |
| Mourning Warbler | MOWA | 107 | 48 | 166 | GROUND | FOL GLEAN | 1.55 | 0.45 |
| Nashville Warbler | NAWA | 330 | 29 | 36 | GROUND | FOL GLEAN | 0.11 | 0.09 |
| Northern Cardinal | NOCA | 72 | 33 | 214 | SHRUB | GROUND | 2.97 | 0.46 |
| Northern Mockingbird | NOMO | 62 | 18 | 82 | SHRUB | GROUND | 1.32 | 0.29 |
| Northern Waterthrush | NOWA | 46 | 8 | 9 | GROUND | GROUND | 0.20 | 0.17 |
| Orchard Oriole | OROR | 16 | 2 | 2 | TREE | FOL GLEAN | 0.13 | 0.13 |
| Ovenbird | OVEN | 574 | 185 | 567 | GROUND | GROUND | 0.99 | 0.32 |
| Red-eyed Vireo | REVI | 206 | 6 | 9 | TREE | FOL GLEAN | 0.04 | 0.03 |
| Red-winged Blackbird | RWBL | 18 | 8 | 18 | SHRUB | GROUND | 1.00 | 0.44 |
| Rose-breasted Grosbeak | RBGR | 109 | 16 | 26 | TREE | FOL GLEAN | 0.24 | 0.15 |
| Savannah Sparrow | SAVS | 16 | 1 | 1 | GROUND | GROUND | 0.06 | 0.06 |
| Scarlet Tanager | SCTA | 20 | 1 | 1 | TREE | FOL GLEAN | 0.05 | 0.05 |
| Slate-colored Junco | SCJU | 29 | 4 | 4 | GROUND | GROUND | 0.14 | 0.14 |
| Song Sparrow | SOSP | 170 | 75 | 312 | SHRUB | GROUND | 1.84 | 0.44 |
| Swainson's Thrush | SWTH | 31 | 7 | 23 | SHRUB | FOL GLEAN | 0.74 | 0.23 |
| Swamp Sparrow | SWSP | 35 | 18 | 73 | SHRUB | GROUND | 2.09 | 0.51 |
| Veery | VEER | 263 | 157 | 764 | GROUND | GROUND | 2.90 | 0.60 |
| White-throated Sparrow | WTSP | 333 | 152 | 546 | GROUND | GROUND | 1.64 | 0.46 |
| Wood Thrush | WOTH | 92 | 37 | 192 | TREE | GROUND | 2.09 | 0.40 |
| Yellow-bellied Chat | YBCH | 17 | 12 | 32 | SHRUB | FOL GLEAN | 1.88 | 0.71 |
